# Quality of patient‐reported outcomes in oncology clinical trials using immune checkpoint inhibitors: A systematic review

**DOI:** 10.1002/cam4.4086

**Published:** 2021-06-29

**Authors:** Eoghan Malone, Reeta Barua, Nicholas Meti, Xuan Li, Rouhi Fazelzad, Aaron R. Hansen

**Affiliations:** ^1^ University of Toronto Toronto ON Canada; ^2^ Division of Medical Oncology and Hematology Princess Margaret Hospital University Health Network Toronto ON Canada; ^3^ Department of Biostatistics Princess Margaret Hospital University Health Network Toronto ON Canada; ^4^ Library and Information Services Princess Margaret Hospital University Health Network Toronto ON Canada

**Keywords:** immune checkpoint inhibitors, patient‐reported outcomes, quality control, systematic review

## Abstract

**Background:**

There are limited data regarding the quality of patient‐reported outcome (PRO) data in immune checkpoint inhibitor (ICI) clinical trial publications.

**Methods:**

A systematic search of citations from various databases was conducted to identify prospective clinical trials involving ICI in advanced tumors from 2003 to 2020. A 30‐point score was adapted from the CONSORT PRO extension statement to assess adherence to CONSORT PRO reporting. Linear regression was used to identify factors associated with quality reporting.

**Results:**

After the review of 8058 articles, 33 trials were included with ICIs as either monotherapy (91%) or part of a combination regimen (9%). The median score was 23.5 points (range 15–29). In the majority of cases (82%), PROs were reported in a separate publication from the original study. Most of the trials were conducted in the metastatic setting and predominantly in melanoma, lung, and renal cell carcinoma (RCC) (73%). Univariate analysis revealed that trials with greater than 250 patients were associated with a higher score. The score was more likely to be lower in disease sites other than melanoma, lung, and RCC and was higher in the KEYNOTE than in the CHECKMATE trial series. There was no significant correlation between the score and whether a trial met its primary end‐point or if the trial improved or worsened the quality of life. In the multivariate analysis, the number of patients enrolled to the trial, disease site, and trial series remained significant.

**Conclusions:**

The quality of reporting of PROs in ICI phase II and III clinical trials is heterogeneous across various cancer sites. As PRO data are increasingly used to counsel patients and complement clinical decision making, innovative and collaborative efforts are required to improve the reporting of these essential data.

## Graphical abstract

There are limited data regarding the quality of patient‐reported outcome data in immune checkpoint inhibitor clinical trial publications. A systematic review was conducted of published clinical trials to assess the quality of reporting.

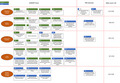

## 1 INTRODUCTION

Evasion of host immune responses and tumor‐mediated immune suppression is an essential component of oncogenesis.^1^ Immune checkpoints are important regulators of the immune system that can be hijacked by cancers to suppress anti‐tumoral T‐cell responses.^2^ Immune checkpoint inhibitors (ICIs) interfere with the tumor's ability to suppress T cells and as a result enhance antitumor immunity.^3^, ^4^ Immune checkpoint therapies are approved for the treatment of a wide range of tumor types with a subset of patients having long‐term durable responses.^4^, ^5^, ^6^, ^7^, ^8^ Among the immune checkpoint pathways, antibody‐mediated blockade of programmed cell death protein‐1 (PD‐1), PD‐1 ligand (PD‐L1), and cytotoxic T‐lymphocyte‐associated protein‐4 (CTLA‐4) have shown the most promising therapeutic outcomes among a variety of cancer types—with other immune checkpoint pathways currently under investigation. In some clinical trials testing ICIs, patient‐reported outcome (PRO) data have been collected to evaluate the risk‐benefit of these agents.

Patient‐reported outcomes are information pertaining to health, quality of life or functional status directly collected from patients at a particular timepoint without processing by clinicians or researchers.^9^ PROs are often used to assess health‐related quality of life (HRQoL), which is a subjective measure of the impact of disease and treatment on a patient's well‐being and can inform the assessment of an intervention's risk‐benefit balance. The use of PROs before, during, and after an intervention can help to measure the impact of that intervention. The utilization of these measures has increased over time due to an increased interest in patient‐centered care and particularly, quality of life.^10^ As PROs are intrinsically subjective and require completion by patients within a specific time frame, they present a range of scientific and logistical challenges for researchers such as incomplete data collection, patient drop‐out, lack of longitudinal follow‐up, and optimal timing of PRO administration in addition to other issues. In order to maximize the full value of PROs, researchers must understand how to incorporate PRO endpoints and assessments into clinical trials, and how to report and interpret PRO information.

Different recommendations have been compiled in order to improve and standardize the use and reporting of PROs in randomized control trials (RCTs). The PROTEUS (PRO tools: engaging users and stakeholders) consortium has provided guidance and recommendations for writing PRO protocols, selecting PRO measures, analyzing, reporting, and interpreting PRO data.^11^ The SPIRIT (Standard Protocol Items: Recommendations for Interventional Trials) PRO extension provides evidence‐based recommendations for the inclusion of PRO content in protocols.^12^ The CONSORT (Consolidated Standards of Reporting Trials) Statement aims to improve the reporting of RCTs but lacks guidance on the reporting of PROs that are often inadequately reported. The CONSORT PRO extension provides guidance to authors of trials describing PROs in order to improve the reporting.^13^ However, adherence to guidelines cannot be presumed and warrants measurement. The objective of this study was to measure the quality of PRO reporting in oncology clinical trials that evaluated ICIs and to explore factors that may affect adherence to the CONSORT PRO extension.

## 2 METHODS

### 2.1 Data sources and searches

**FIGURE 1:**
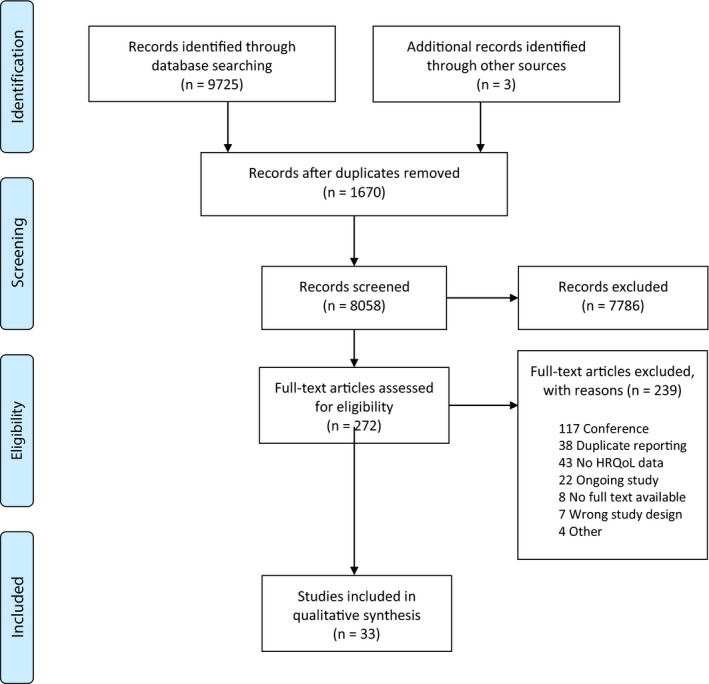
Selection strategy—study inclusion and exclusion flowchart based on Preferred Reporting Items for Systematic Reviews and Meta‐analyses

We followed the Preferred Reporting Items for Systematic Reviews and Meta‐Analyses guideline (Figure 1).^14^ A systematic literature search for studies meeting pre‐defined criteria was performed in the following databases: Medline (Appendix A), Epub Ahead of Print and In‐Process & Other Non‐indexed Citations, Cochrane Central Register Trials, Cochrane Database of Systematic Reviews, Embase, and PsycInfo all from the OvidSp platform; where provided, both controlled vocabulary terms and text words were used; there were no language or type of study restriction. The time period for the search ranged from January 1^st^, 2003 as starting date and the end of study inclusion was March 1, 2020. The search strategy used a combination of terms (including controlled vocabulary—e.g., MeSH [Medical Subject Headings]) and text words developed in consultation with an oncology liaison research librarian. Existing systematic reviews on this topic will also be analyzed and cross‐referenced to identify any additional clinical trials.

### 2.2 Study selection

Phase 2 and 3 clinical trials that evaluated ICIs (ipilimumab, nivolumab, pembrolizumab, durvalumab, atezolizumab, and avelumab) for patients with various types of solid and hematological cancers were included. We excluded non‐English language articles, abstracts, case reports, case series, reviews, meta‐analyses, editorials, commentaries, letters, and conference proceedings.

### 2.3 Data extraction

Following a comprehensive literature search and removal of duplicates, we (N.M., R.B., and E.M.) independently reviewed the titles and abstracts of the articles and further screened their full text against our inclusion and exclusion criteria. We extracted the study information and details using a standardized spreadsheet (Microsoft Excel; Microsoft Group). Disagreements during study selection and data extraction were resolved by consensus. The following data were extracted from each eligible article: year of publication, journal, impact factor, number of participants, disease type, treatment setting, phase of study, ICI target and agent used, number of patients, funding, QoL tool used, study results, and funding or study sponsorship.

### 2.4 PRO reporting score

For the purposes of this analysis, a 30‐point scoring system adapted from a previous publication^15^ was developed using the CONSORT PRO checklist (Appendix B). Although the CONSORT and CONSORT PRO extensions were developed for randomized clinical trials, the standards for adverse event (AE) reporting are applicable across all types of clinical trials and for all types of AEs. There are a total of 42 items in the 30‐point scoring system, addressing different domains of clinical trial reporting; 25 points were allocated to the CONSORT reporting criteria and five total points were allocated to the CONSORT PRO extension. Eighteen of the 42 items had a maximum score of one point for complete reporting; all PRO extension items (five total) were included in this full point‐scoring. Some of the CONSORT sections were divided into parts A and B as per the original publication and therefore a value of 0.5 was attributed to these to maintain the 25 point CONSORT system developed (Figure 3)

### 2.5 Statistical analysis

Each article was given three scores (1) overall CONSORT PRO (CP); (2) CONSORT alone (C); and (3) PRO extension (P) scores. The scores were summarized using descriptive statistics. Univariate analyses and multivariate linear regressions were carried out to identify factors associated with higher scores. A *p* ≤ 0.05 was considered statistically significant for all tests. Correlation between CP, C, and P was determined by the Pearson correlation coefficient. A *p* ≤ 0.05 was indicated that all correlations were significantly different from zero.

## 3 RESULTS

### 3.1 Literature search

The literature search yielded 9725 articles. Our research team conducted a review of known systematic reviews on this topic and cross‐referenced clinical trials identified in our search strategy and identified three additional clinical trials to include in our analysis. (Figure 1). After the removal of duplicates, 8058 records remained. Two hundred and seventy‐two titles met the criteria for full‐text review and 33 studies ultimately met full inclusion criteria.

### 3.2 Study characteristics

The majority of studies (*n* = 27, 82%) reported PROs in a separate publication from the original study (Table 1). All of the primary studies were published in journals with impact factors greater than 20. The impact factor of the journals that published PRO publications was greater than 10 in 21 (75%) of the articles, of note journals did not stipulate that submissions must adhere to the CONSORT PRO. The majority of studies were phase 3 RCTs (*n* = 27, 82%) that had enrolled over 500 patients (*n* = 20, 61%). Anti‐PD1/PD‐L1 agents (27/33) were the most common ICI used (*n* = 27, 82%). The majority of the trials were performed in patients with lung cancer, melanoma or renal cell carcinoma (RCC) (*n* = 24, 73%) in the metastatic setting (*n* = 31, 94%). In 29/30 of the phase III studies, there was a significant difference in the primary endpoint of the intervention versus the control arm. Quality of life remained the same or improved throughout the treatment in most clinical trials (*n* = 28, 85%) with ICIs. PRO reporting was performed using a variety of tools, and certain studies employed multiple tools. Twenty‐two studies used the EORTC QLQ‐C30 (Quality of Life Questionnaire Core 30) tool, eighteen studies used the EQ‐5D (EuroQol‐5 Dimensions) tool, three studies used the FACT‐M (Functional Assessment of Cancer Therapy—Melanoma) tool and fourteen studies used other tools.

**TABLE 1:** Study characteristics

Study characteristic	No. of studies (*n* = 33)	Mean score (SD)
Method of PRO reporting		
Initial publication	6	21.5 (4)
PRO publication	27	23.5 (3.2)
Year of primary publication		
≤2016	11	24 (2.4)
>2016	22	22.7 (3.7)
Year of PRO publication		
≤2017	10	24.2 (2.2)
>2017	17	23 (3.6)
IF of PRO publication		
<5	3	20.5 (0.5)
5–10	5	23.9 (1.8)
>10	17	24 (3.7)
No. of patients enrolled in the trial		
≤250	7	18.6 (2.2)
>250	26	24.3 (2.5)
Disease site		
Lung	11	24.1 (3.5)
Melanoma	8	23.2 (2.3)
Renal cell carcinoma	5	25 (2.6)
Other	9	20.8 (3.6)
Treatment intent		
Curative	2	26 (0)
Palliative	31	22.9 (3.4)
Immune therapy target		
PD1/PDL‐1	27	22.9 (3.6)
CTLA4	3	24.7 (3.2)
PD1/PDL‐1 and CTLA4	3	23.2 (1.5)
Trial series		
KEYNOTE	8	25 (3.1)
CHECKMATE	13	21.7 (3.2)
Other	12	23.5 (3.2)
Primary endpoint		
Overall survival	11	23.1 (2.2)
Other	22	23.1 (3.9)
Trial design		
Phase II	6	18.9 (2.2)
Phase III	27	24.1 (2.8)
Primary endpoint met?		
No	1	23 (NA)
Yes	29	23.5 (3.4)
N/A	3	19.7 (1.5)
Q of L assessment outcome		
No change	13	23.1 (2.3)
Better	15	24.1 (3.6)
Worse	2	23 (5.7)
Single‐arm trial	3	18.5 (2.3)

Abbreviation: PRO, patient‐reported outcome.

### 3.3 Individual quality score outcomes

**FIGURE 2:**
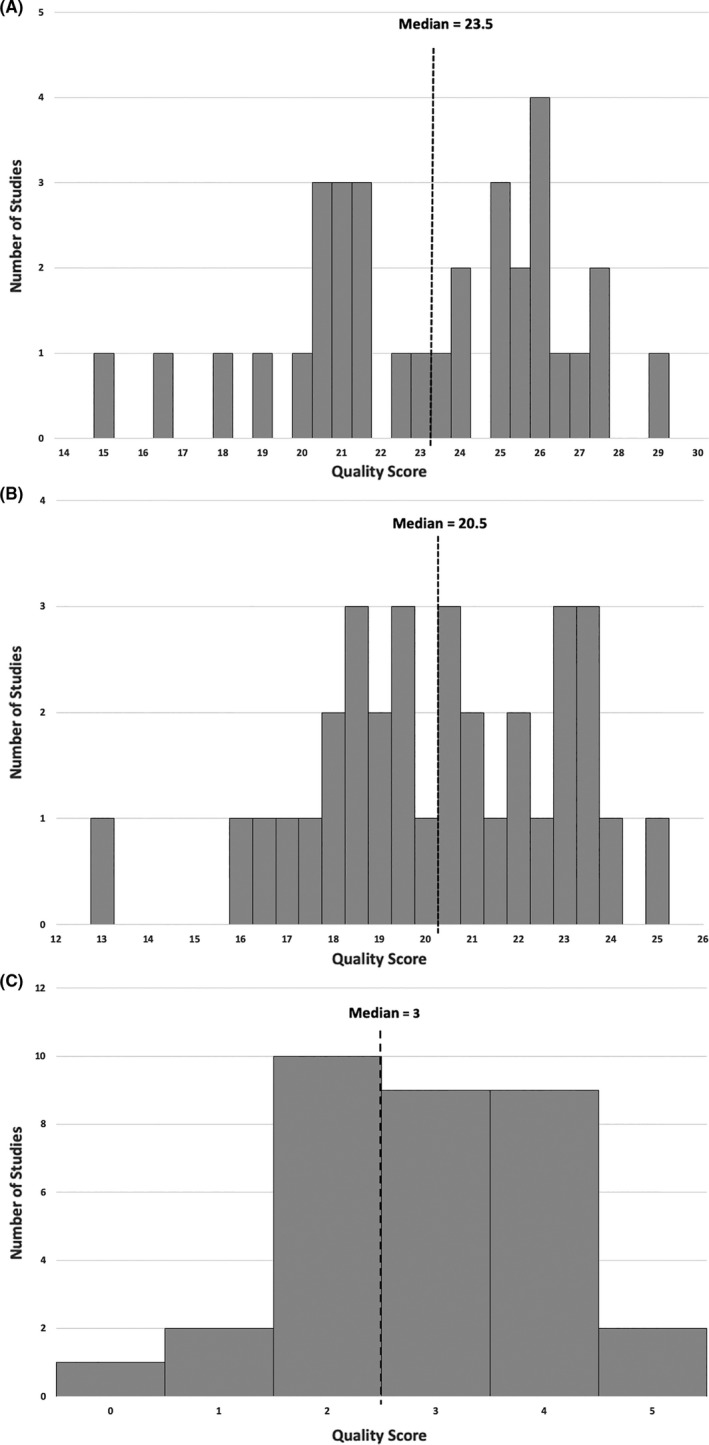
(A) Histogram showing the distribution of the overall quality (Standard CONSORT + PRO‐specific criteria) scores of the 33 immune checkpoint inhibitor clinical trials. Maximum score = 30. (B) Histogram showing the distribution of the quality scores of the 33 immune checkpoint inhibitor clinical trials calculated using the CONSORT 2010 checklist. Maximum score = 25. (C) Histogram showing the distribution of the PRO‐specific quality scores of the 33 immune checkpoint inhibitor clinical trials. Maximum score = 5. CONSORT, Consolidated Standards of Reporting Trials; PRO, patient‐reported outcome

The median CP score among the 33 studies was 23.5 (SD = 3.4) out of 30, with the full distribution of scores shown in Figure 2A. Likewise, the median C and P scores were 20.5 (SD = 2.7) and 3 (SD = 1.2), respectively, with the distributions shown in Figure 2B,C. Phase II studies were more likely to have a lower mean CP score than phase III studies.

**FIGURE 3:**
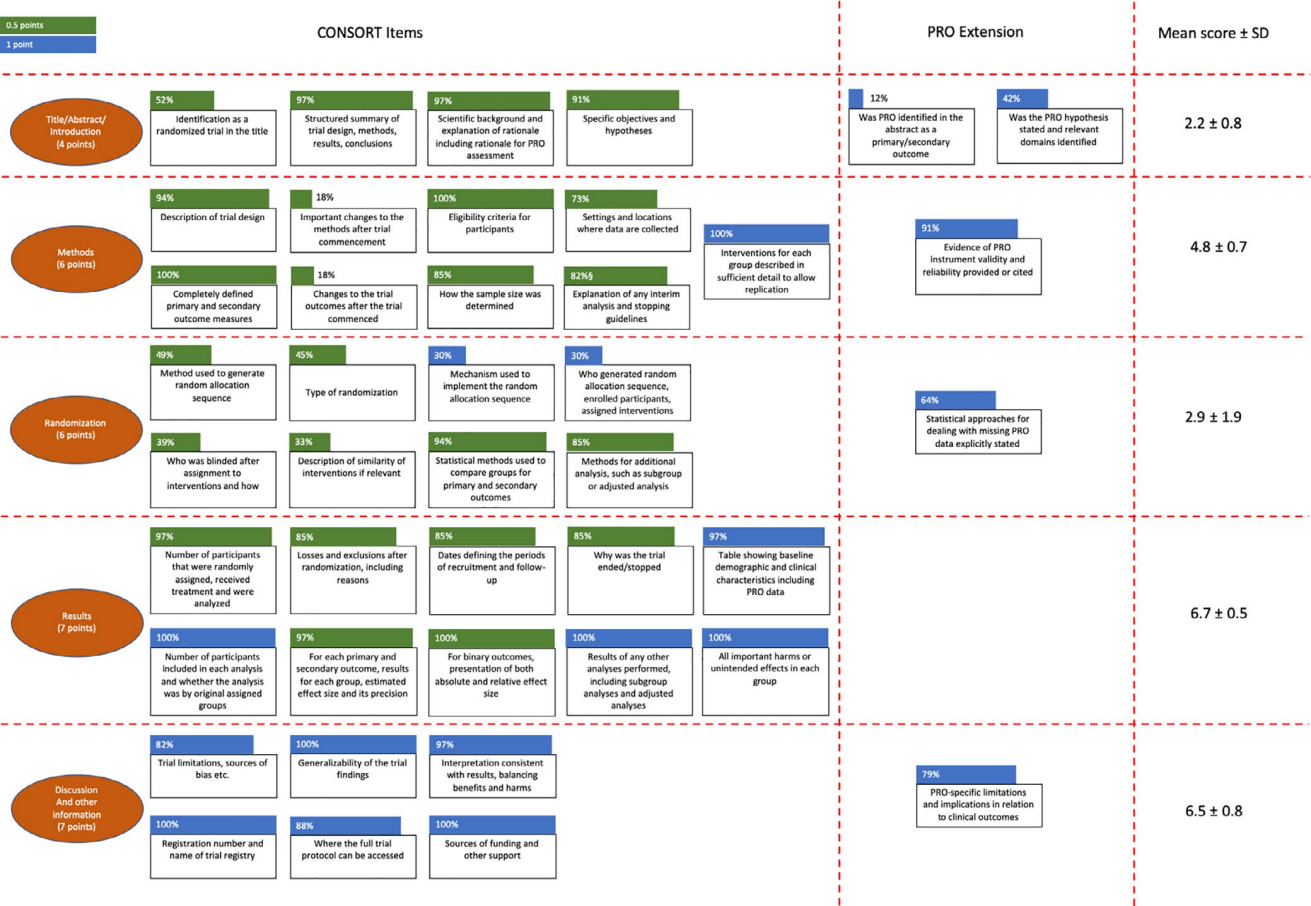
Components of the CONSORT checklist relevant to patient‐reported outcomes, and the scoring of each item from the 33 clinical trials. CONSORT, Consolidated Standards of Reporting Trials

Figure 3 shows a complete summary of the scoring of all items in the CONSORT PRO checklist. PROs were identified in the abstracts of the initial publications as primary or secondary outcomes only 12% of the time, and a clear PRO‐related hypothesis was provided 42% of the time. The background and rationale for performing a PRO assessment were stated 96% of the time, and adequate evidence of the PRO instrument validity was provided 91% of the time. The results of the PRO analyses were reported appropriately over 90% of the time, although PRO‐specific limitations were only addressed 79% of the time.

### 3.4 Univariate and multivariate analyses

Univariate analysis showed that trials with greater than 250 patients were associated with a higher quality score (Table 2). The quality score was more likely to be lower in trials that were conducted in disease sites other than lung, melanoma or RCC. CP scores were also more likely to be lower in the CHECKMATE clinical trial series versus the KEYNOTE clinical trial series. Phase III studies were more likely to have a higher quality score than phase II trials. Notably, there was no significant correlation between the quality score and whether the trial met its primary endpoint or if the trial improved or worsened quality of life. In multivariate analysis, the number of patients enrolled in the trial, disease site, and trial series all remained significant (Table 3).

**TABLE 2:** Univariate analysis

	Coefficient	95% Confidence interval	*p*‐value
Method of PRO reporting			
Initial publication	Reference		
PRO publication	1.98	(−0.97, 4.93)	0.19
Year of primary publication			
≤2016	Reference		
>2016	−1.39	(−3.82, 1.04)	0.26
Year of PRO publication			
≤2017	Reference		
>2017	−1.22	(−3.72, 1.28)	0.34
IF of PRO publication			
<5	Reference		
5–10	3.4	(−1.21, 8.01)	0.16
>10	3.53	(−0.42, 7.48)	0.094
No. of patients enrolled in the trial			
≤250	Reference		
>250	5.77	(3.77, 7.78)	<0.001
Disease site			
Lung	Reference		
Melanoma	−0.95	(−3.8, 1.91)	0.52
Renal cell carcinoma	0.86	(−2.45, 4.18)	0.61
Other	−3.36	(−6.12, −0.6)	0.024
Treatment intent			
Curative	Reference		
Palliative	−3.06	(−7.84, 1.71)	0.21
Immune therapy target			
PD1/PDL‐1	Reference		
CTLA4	1.72	(−2.38, 5.83)	0.42
PD1/PDL‐1 and CTLA4	0.22	(−3.88, 4.33)	0.92
Trial series			
KEYNOTE	Reference		
CHECKMATE	−3.35	(−6.16, −0.53)	0.027
Other	−1.54	(−4.4, 1.32)	0.3
Primary endpoint			
Overall survival	Reference		
Other	−0.02	(−2.5, 2.46)	0.99
Trial design			
Phase II	Reference		
Phase III	5.14	(2.71, 7.57)	<0.001
Primary endpoint met?			
N	Reference		
Y	0.48	(−6.07, 7.03)	0.89
N/A	−3.33	(−10.77, 4.1)	0.39
Quality of life assessment outcome			
No change	Reference		
Better	0.95	(−1.38, 3.28)	0.43
Worse	−0.12	(−4.79, 4.56)	0.96
Single‐arm trial	−4.62	(−8.56, −0.67)	0.029

**TABLE 3:** Multivariate analysis

Covariate	Estimate (95% CI)	*p*‐value	Global *p*‐value
Number of patients enrolled in the trial			**<0.001**
≤250	Reference		
>250	5.64 (3.63,7.65)		
Disease site			0.062
Lung	Reference		
Melanoma	−0.94 (−2.82,0.94)	0.34	
Renal cell carcinoma	2.21 (−0.02,4.44)	0.063	
Other	−0.26 (−2.29,1.65)	0.8	
Trial series			0.0048
KEYNOTE	Reference		
CHECKMATE	−3.02 (−4.86,−1.18)	0.0035	
Other	−1.47 (−3.36,0.43)	0.14	

### 3.5 Correlation between CP, C, and P scores

In all studies, there was a strong correlation between C and CP scores, with a correlation coefficient of 0.95 (*p* < 0.01). There was a moderate correlation between CP and P scores, with a coefficient of 0.7 (*p* < 0.01). Last, there was a weak correlation between C and P scores, correlation coefficient 0.44 (*p* = 0.01). Six studies reported PROs in the initial trial publication. Among these six studies, the correlation coefficient between C and CP scores was 0.99 (*p* < 0.01). The correlation coefficient between P and CP scores was 0.91 (*p* = 0.01). Last, the correlation coefficient between C and P scores was 0.84 (*p* = 0.04). The remaining 27 studies reported PROs in a separate publication. Among these studies, the correlation coefficient between C and CP scores was 0.96 (*p* < 0.01). The correlation coefficient between P and CP scores was 0.65 (*p* < 0.01). Last, the correlation coefficient between C and P scores was 0.42 (*p* = 0.03).

## 4 DISCUSSION

We report the largest systematic review of PRO data from clinical trials of ICIs and describe for the first time the adherence of publications to the CONSORT PRO extension guideline. The scoring system used in this study was based on the CONSORT PRO and adapted a previously developed AE reporting score for investigating AE^16^ and immune‐related AE reporting^15^ in clinical trials. Of note, there were no requirements laid out by any of the journals on how to report PROs. The scores demonstrate that the majority of studies accounted for a high number of the items in the CONSORT PRO extension, which implies that the reporting in these trials was of above‐average quality. Several factors were associated with high scores including the number of patients enrolled on the trial, tumor type, and agent tested. However, none of these trials had a PRO or HRQOL as a primary or secondary endpoint and none of these studies had an HRQOL hypothesis a priori. Thus, while we have demonstrated that the quality of reporting of PRO data in these studies is excellent, it is clear that there is a need to design the clinical trials of ICIs with a primary or secondary HRQOL endpoint and ensure that they are adequately powered to demonstrate changes as measured by the chosen PRO instrument.

Immune checkpoint inhibitors have a unique set of immune‐mediated toxicities, which need to be described adequately, accurately, and in a standardized way to ensure balanced reporting of treatment benefits and associated toxicity profiles. In clinical trials, AEs have been traditionally graded by the physician. However, there is a role for patients reporting their experiences of treatment to gain a more complete understanding of the ramifications of treatment. The trials included in this study largely used tools measuring HRQOL rather than health status. Although PROs are commonly included in cancer clinical trial protocols, heterogeneous PRO data reporting has made interpreting these information difficult. For example, Faury et al have shown that the reporting of HRQOL data in ICI clinical trials is inconsistent, in addition to the lack of pre‐defined hypotheses about how ICI agents would impact HRQOL.^17^ Other analyses have shown significant heterogeneity in PRO instruments used in data collection.^18^ These studies highlight the need to identify improved methods and standards in obtaining and reporting high‐quality, standardized PRO data. Our systematic review was designed to complement this work by quantifying the quality of PRO reporting using existing guidelines.

The assessment of the scores was based on the review of the full trial publication including any Supporting Information appendices. Our findings were that journals with a higher impact factor were associated with better reporting standards of ICIs. Increased rigor of editorial review and more stringent publication requirements may account for these differences, although it is notable that no journal required publications to adhere to the CONSORT PRO guidelines. In the multivariate analysis, the number of patients enrolled in the trial, disease site, and trial series were independent factors associated with higher scores. Supposedly larger studies (which were typically performed in melanoma, NSCLC, and RCC populations) may have had more resources to collect PRO data and subsequently report this information. Furthermore, publication bias and sample size bias^19^, ^20^ may have also influenced this observation with smaller studies having less positive or citable results, and thus they were less likely to be published. It is unclear why there would be a difference in scores between CHECKMATE and KEYNOTE trials; however, this may relate to internal publication procedures and standards, for example, the use of different medical writing services between these sponsors. We do not assert that there is any difference in HRQOL between the KEYNOTE and CHECKMATE trials. There was no significant correlation between the score and whether a trial met its primary end‐point or if the trial improved or worsened the HRQOL. This is most likely due to the small number of studies in this review and the fact that most studies meet their stated primary objective. It is also reassuring to note that the score was not associated with positive or negative changes in HRQOL, meaning that reporting was of good standard regardless of these results. There was a weak correlation between C and P scores, which could be related to the RCT and the PRO data being reported in separate articles.

There are ongoing international initiatives to improve the utility of PROs in clinical trials. For instance, the International Society for Quality Of Life research is a global community of researchers, clinicians, health care professionals, industry professionals, consultants, and research partners advancing HRQoL in order to ensure that the patient perspective is integral to health research, care, and policy.^21^ The PROTEUS consortium (PRO tools: engaging users and stakeholders) engages key stakeholders to promote the application of tools developed to optimize the assessment and reporting of PROs in clinical trials.^11^ The SISAQOL (Setting International Standards in Analyzing PROs and Quality of Life Endpoints Data) is an international consortium directed by the EORTC to provide recommendations to standardize the analysis of HRQOL and other PRO data in cancer RCTs.^22^ SISAQOL has proposed that the areas of priority include identifying appropriate statistical methods for analysis, standardizing terminology related to missing data, and managing missing data.^23^ Following SPIRIT and SPIRIT‐PRO guidance should improve the quality of data produced by clinical trials and inform patient‐centered care.^12^

The EQUATOR network (Enhancing the QUAlity and Transparency Of health Research) is an international initiative that seeks to improve the reliability and value of published research literature by promoting transparent and accurate reporting and wider use of robust reporting guidelines.^24^ Its aim is to raise awareness regarding the importance of good reporting of research, assisting in the development, and implementation of reporting guidelines. It is an umbrella organization bringing together the developers of reporting guidelines, medical journal editors, peer reviewers, and other stakeholders to improve the quality of research publications and the research itself. Protocol developers, particularly those not familiar with PRO methodology, may benefit from the explanation of PRO‐specific aspects to facilitate improvements in the content.

There are several limitations to our study. The conclusions must be interpreted in the context of a relatively small number of trials that were included because the estimated coefficients and p‐values might be not reliable. The scoring system measures the adequacy of reporting the results, and as such, this study did not assess the actual PRO data. In our study, we included the primary paper and the PRO paper if they were reported separately which may have inflated the scores. However, given that items in the CONSORT PRO checklist pertain to both the RCT and PRO data, all relevant information published by the trial investigators should be eligible for inclusion.

## 5 CONCLUSION

Our systematic review has demonstrated that the reporting quality of PRO data in RCTs of ICIs is of good to high standard. Larger studies and cancer type (melanoma, NSCLC, and RCC) were independently associated with better quality reporting. Achieving the primary objective or changes in HRQOL did not affect the quality of reporting. Many initiatives are underway to enhance PRO integration into trial design and operation, and PRO reporting and interpretation.

## CONFLICT OF INTEREST

Dr. Eoghan Malone reports no conflicts of interest; Dr. Reeta Barua reports no conflicts of interest; Dr. Nicholas Meti reports no conflicts of interest; Dr. Aaron R. Hansen: consulting and research support from Genetech Inc., Hoffmann La Roche Inc., Merck Serono S.A., GlaxoSmithKline Inc., Bristol‐Myers Squibb Company, Novartis Pharmaceuticals Canada Inc., Boston Biomedical Inc., Boehringer Ingelheim International GmbH, AstraZeneca Pharmaceuticals LP, MedImmune LL, Pfizer Inc.

## AUTHOR CONTRIBUTION

Concept and design: EM, RB, and AH; Financial support: N/A; Collection and assembly of data: EM, RB, and NM; Data analysis and interpretation: All authors; Manuscript writing: All authors; Final approval of the manuscript: All authors.

## ETHICAL APPROVAL

Ethical approval was not required for this review article.

## Supporting information

Supplementary Material

Supplementary Material

## Data Availability

Database of information retrieved from articles analyzed available upon request.
