# Supplementary material for: Quality of patient‐reported outcomes in oncology clinical trials using immune checkpoint inhibitors: A systematic review
Source: Cancer Med. 2021 Jun 29;10(15):5031–40. doi: 10.1002/cam4.4086 (PMC8335827; doi:10.1002/cam4.4086)
Supplement: Supplementary file 1 — Supplementary Material [file CAM4-10-5031-s001.docx]

**Appendix A**

Literature search strategy

The following databases were searched:

- Medline
- Epub Ahead of Print and In-Process & Other Non-indexed Citations
- Cochrane Central Register Trials
- Cochrane Database of Systematic Reviews
- Embase
- PsycInfo

all from the OvidSp

Search terms

**Ovid MEDLINE(R) 2003 to March 12, 2020**

| **#** | **Searches** | **Results** | **Type** |  |  |  |
| --- | --- | --- | --- | --- | --- | --- |
|  | | | | | | |
| 1 | exp Neoplasms/ | 3294993 | Advanced |  |  |  |
| 2 | Cancer Survivors/ | 3026 | Advanced |  |  |  |
| 3 | neoplas*.mp,kw. | 2827600 | Advanced |  |  |  |
| 4 | paraneoplas*.mp,kw. | 12642 | Advanced |  |  |  |
| 5 | cancer*.mp,kw. | 1514918 | Advanced |  |  |  |
| 6 | tumo?r*.mp,kw. | 1883296 | Advanced |  |  |  |
| 7 | onco*.mp,kw. | 474610 | Advanced |  |  |  |
| 8 | metast*.mp,kw. | 487469 | Advanced |  |  |  |
| 9 | malignan*.mp,kw. | 498590 | Advanced |  |  |  |
| 10 | aberrant crypt foci.mp,kw. | 1465 | Advanced |  |  |  |
| 11 | acanthoma*.mp,kw. | 698 | Advanced |  |  |  |
| 12 | acrospiroma*.mp,kw. | 548 | Advanced |  |  |  |
| 13 | adamantinom*.mp,kw. | 980 | Advanced |  |  |  |
| 14 | adenocarc*.mp,kw. | 218538 | Advanced |  |  |  |
| 15 | adenofibrom*.mp,kw. | 2299 | Advanced |  |  |  |
| 16 | adenolymphom*.mp,kw. | 1734 | Advanced |  |  |  |
| 17 | adenomat*.mp,kw. | 21304 | Advanced |  |  |  |
| 18 | adenomyo*.mp,kw. | 3439 | Advanced |  |  |  |
| 19 | adenosarcom*.mp,kw. | 564 | Advanced |  |  |  |
| 20 | adenosquam*.mp,kw. | 3382 | Advanced |  |  |  |
| 21 | ameloblastom*.mp,kw. | 4163 | Advanced |  |  |  |
| 22 | androblastom*.mp,kw. | 327 | Advanced |  |  |  |
| 23 | angiofibrom*.mp,kw. | 2116 | Advanced |  |  |  |
| 24 | angiokeratom*.mp,kw. | 1091 | Advanced |  |  |  |
| 25 | angiolipom*.mp,kw. | 607 | Advanced |  |  |  |
| 26 | angioma*.mp,kw. | 12452 | Advanced |  |  |  |
| 27 | angiomyolipom*.mp,kw. | 3724 | Advanced |  |  |  |
| 28 | angiomyom*.mp,kw. | 586 | Advanced |  |  |  |
| 29 | angiosarcom*.mp,kw. | 5491 | Advanced |  |  |  |
| 30 | apudoma*.mp,kw. | 559 | Advanced |  |  |  |
| 31 | arrhenoblastom*.mp,kw. | 348 | Advanced |  |  |  |
| 32 | astrocytom*.mp,kw. | 20672 | Advanced |  |  |  |
| 33 | blastom*.mp,kw. | 10395 | Advanced |  |  |  |
| 34 | Bowen*.mp,kw. | 3229 | Advanced |  |  |  |
| 35 | Brenner*.mp,kw. | 1427 | Advanced |  |  |  |
| 36 | Buschke-Lowenstein*.mp,kw. | 252 | Advanced |  |  |  |
| 37 | carcin*.mp,kw. | 933953 | Advanced |  |  |  |
| 38 | cementoma*.mp,kw. | 494 | Advanced |  |  |  |
| 39 | chemodectomas*.mp,kw. | 298 | Advanced |  |  |  |
| 40 | cholangiocarcin*.mp,kw. | 12015 | Advanced |  |  |  |
| 41 | chondroblastom*.mp,kw. | 1316 | Advanced |  |  |  |
| 42 | chondroma*.mp,kw. | 6237 | Advanced |  |  |  |
| 43 | chordoma*.mp,kw. | 4087 | Advanced |  |  |  |
| 44 | chondrosarcom*.mp,kw. | 8963 | Advanced |  |  |  |
| 45 | choriocarcin*.mp,kw. | 9002 | Advanced |  |  |  |
| 46 | craniopharyngioma*.mp,kw. | 5249 | Advanced |  |  |  |
| 47 | cystadenofibrom*.mp,kw. | 213 | Advanced |  |  |  |
| 48 | cystosarcom*.mp,kw. | 614 | Advanced |  |  |  |
| 49 | cytoma*.mp,kw. | 326 | Advanced |  |  |  |
| 50 | dermatofibrosarcom*.mp,kw. | 1887 | Advanced |  |  |  |
| 51 | desmoplas*.mp,kw. | 4062 | Advanced |  |  |  |
| 52 | dysgerminoma*.mp,kw. | 6068 | Advanced |  |  |  |
| 53 | DCIS.mp,kw. | 4311 | Advanced |  |  |  |
| 54 | DSRCT.mp,kw. | 255 | Advanced |  |  |  |
| 55 | ependymom*.mp,kw. | 6537 | Advanced |  |  |  |
| 56 | Ewing*.mp,kw. | 9889 | Advanced |  |  |  |
| 57 | fibroadenom*.mp,kw. | 4040 | Advanced |  |  |  |
| 58 | fibroepithelial*.mp,kw. | 941 | Advanced |  |  |  |
| 59 | fibroma*.mp,kw. | 18398 | Advanced |  |  |  |
| 60 | fibrosarcom*.mp,kw. | 16419 | Advanced |  |  |  |
| 61 | FAMMM.mp,kw. | 71 | Advanced |  |  |  |
| 62 | gangliogliom*.mp,kw. | 1506 | Advanced |  |  |  |
| 63 | ganglioneurom*.mp,kw. | 3126 | Advanced |  |  |  |
| 64 | gastrinoma*.mp,kw. | 1835 | Advanced |  |  |  |
| 65 | germinoma*.mp,kw. | 3882 | Advanced |  |  |  |
| 66 | glioblastom*.mp,kw. | 35542 | Advanced |  |  |  |
| 67 | glioma*.mp,kw. | 56878 | Advanced |  |  |  |
| 68 | gliosarcom*.mp,kw. | 1176 | Advanced |  |  |  |
| 69 | glomus jugulare*.mp,kw. | 1363 | Advanced |  |  |  |
| 70 | glomus tympanicum*.mp,kw. | 259 | Advanced |  |  |  |
| 71 | glucagonoma*.mp,kw. | 1118 | Advanced |  |  |  |
| 72 | gonadoblastom*.mp,kw. | 793 | Advanced |  |  |  |
| 73 | GCTOB.mp,kw. | 6 | Advanced |  |  |  |
| 74 | GIST?.mp,kw. | 6294 | Advanced |  |  |  |
| 75 | hemangioendotheliom*.mp,kw. | 4164 | Advanced |  |  |  |
| 76 | hemangiom*.mp,kw. | 32596 | Advanced |  |  |  |
| 77 | hemangiopericytom*.mp,kw. | 3558 | Advanced |  |  |  |
| 78 | hemangiosarcom*.mp,kw. | 7299 | Advanced |  |  |  |
| 79 | hamartoblastom*.mp,kw. | 48 | Advanced |  |  |  |
| 80 | hepatoblastom*.mp,kw. | 3444 | Advanced |  |  |  |
| 81 | hepatoma*.mp,kw. | 27250 | Advanced |  |  |  |
| 82 | histiocytom*.mp,kw. | 8416 | Advanced |  |  |  |
| 83 | hodgkin*.mp,kw. | 86083 | Advanced |  |  |  |
| 84 | nonhodgkin*.mp,kw. | 114 | Advanced |  |  |  |
| 85 | (hutchinson* adj2 freckle*).mp,kw. | 706 | Advanced |  |  |  |
| 86 | HNPCC.mp,kw. | 2124 | Advanced |  |  |  |
| 87 | immunocytom*.mp,kw. | 595 | Advanced |  |  |  |
| 88 | incidentaloma?.mp,kw. | 1790 | Advanced |  |  |  |
| 89 | insulinoma*.mp,kw. | 7201 | Advanced |  |  |  |
| 90 | kasabach merrit*.mp,kw. | 711 | Advanced |  |  |  |
| 91 | leiomyoblastom*.mp,kw. | 392 | Advanced |  |  |  |
| 92 | leiomyom*.mp,kw. | 22858 | Advanced |  |  |  |
| 93 | leiomyosarcom*.mp,kw. | 11197 | Advanced |  |  |  |
| 94 | leukem*.mp,kw. | 298753 | Advanced |  |  |  |
| 95 | preleukem*.mp,kw. | 2048 | Advanced |  |  |  |
| 96 | leukoplak*.mp,kw. | 6223 | Advanced |  |  |  |
| 97 | li-fraumeni*.mp,kw. | 1270 | Advanced |  |  |  |
| 98 | lipoblastom*.mp,kw. | 454 | Advanced |  |  |  |
| 99 | lipoma*.mp,kw. | 17733 | Advanced |  |  |  |
| 100 | liposarcom*.mp,kw. | 6621 | Advanced |  |  |  |
| 101 | luteoma*.mp,kw. | 219 | Advanced |  |  |  |
| 102 | lymphangio*.mp,kw. | 14415 | Advanced |  |  |  |
| 103 | lymphoblastom*.mp,kw. | 362 | Advanced |  |  |  |
| 104 | lymphocytom*.mp,kw. | 329 | Advanced |  |  |  |
| 105 | lymphoma*.mp,kw. | 224114 | Advanced |  |  |  |
| 106 | lymphosarcom*.mp,kw. | 5074 | Advanced |  |  |  |
| 107 | lynch*.mp,kw. | 3456 | Advanced |  |  |  |
| 108 | macroglobulinem*.mp,kw. | 5937 | Advanced |  |  |  |
| 109 | m?croprolactinom*.mp,kw. | 732 | Advanced |  |  |  |
| 110 | mastocytom*.mp,kw. | 2025 | Advanced |  |  |  |
| 111 | mastocytos?s*.mp,kw. | 3970 | Advanced |  |  |  |
| 112 | medulloblastom*.mp,kw. | 8955 | Advanced |  |  |  |
| 113 | meigs*.mp,kw. | 852 | Advanced |  |  |  |
| 114 | melanoameloblastom*.mp,kw. | 11 | Advanced |  |  |  |
| 115 | melanoblastom*.mp,kw. | 490 | Advanced |  |  |  |
| 116 | melanocarcin*.mp,kw. | 101 | Advanced |  |  |  |
| 117 | melanoma*.mp,kw. | 119516 | Advanced |  |  |  |
| 118 | melanosis.mp,kw. | 4190 | Advanced |  |  |  |
| 119 | melanotic*.mp,kw. | 2977 | Advanced |  |  |  |
| 120 | meningiom*.mp,kw. | 23052 | Advanced |  |  |  |
| 121 | mesenchymom*.mp,kw. | 2079 | Advanced |  |  |  |
| 122 | mesoblast*.mp,kw. | 745 | Advanced |  |  |  |
| 123 | mesonephrom*.mp,kw. | 1165 | Advanced |  |  |  |
| 124 | mesotheliom*.mp,kw. | 16771 | Advanced |  |  |  |
| 125 | metaplas*.mp,kw. | 22721 | Advanced |  |  |  |
| 126 | micrometast*.mp,kw. | 6205 | Advanced |  |  |  |
| 127 | muir-torre*.mp,kw. | 411 | Advanced |  |  |  |
| 128 | myelolipom*.mp,kw. | 998 | Advanced |  |  |  |
| 129 | myoepitheliom*.mp,kw. | 1284 | Advanced |  |  |  |
| 130 | myofibrom*.mp,kw. | 750 | Advanced |  |  |  |
| 131 | myeloma*.mp,kw. | 55681 | Advanced |  |  |  |
| 132 | myoma*.mp,kw. | 5874 | Advanced |  |  |  |
| 133 | myosarcom*.mp,kw. | 403 | Advanced |  |  |  |
| 134 | myxofibrosarcom*.mp,kw. | 432 | Advanced |  |  |  |
| 135 | myxoma*.mp,kw. | 9720 | Advanced |  |  |  |
| 136 | myxosarcom*.mp,kw. | 467 | Advanced |  |  |  |
| 137 | n?evocarcin*.mp,kw. | 78 | Advanced |  |  |  |
| 138 | neurilemmom*.mp,kw. | 13739 | Advanced |  |  |  |
| 139 | neurocytom*.mp,kw. | 808 | Advanced |  |  |  |
| 140 | neuroectodermal*.mp,kw. | 7982 | Advanced |  |  |  |
| 141 | neurofibroma*.mp,kw. | 19733 | Advanced |  |  |  |
| 142 | neurofibrosarcom*.mp,kw. | 524 | Advanced |  |  |  |
| 143 | neurilemmom*.mp,kw. | 13739 | Advanced |  |  |  |
| 144 | neuroblastom*.mp,kw. | 39157 | Advanced |  |  |  |
| 145 | neuroma*.mp,kw. | 13887 | Advanced |  |  |  |
| 146 | neurothekeom*.mp,kw. | 257 | Advanced |  |  |  |
| 147 | NSCLC.mp,kw. | 32381 | Advanced |  |  |  |
| 148 | odontoma*.mp,kw. | 1431 | Advanced |  |  |  |
| 149 | oligodendrogliom*.mp,kw. | 5232 | Advanced |  |  |  |
| 150 | oligometast*.mp,kw. | 1162 | Advanced |  |  |  |
| 151 | osteoblastom*.mp,kw. | 1177 | Advanced |  |  |  |
| 152 | osteochondrom*.mp,kw. | 3020 | Advanced |  |  |  |
| 153 | osteoclastom*.mp,kw. | 329 | Advanced |  |  |  |
| 154 | osteoma*.mp,kw. | 13252 | Advanced |  |  |  |
| 155 | osteosarcom*.mp,kw. | 27695 | Advanced |  |  |  |
| 156 | papilloma*.mp,kw. | 64881 | Advanced |  |  |  |
| 157 | papillary*.mp,kw. | 61188 | Advanced |  |  |  |
| 158 | paragangliom*.mp,kw. | 8069 | Advanced |  |  |  |
| 159 | pheochromocytom*.mp,kw. | 20894 | Advanced |  |  |  |
| 160 | phyllo?des*.mp,kw. | 2231 | Advanced |  |  |  |
| 161 | pinealocytoma*.mp,kw. | 16 | Advanced |  |  |  |
| 162 | pinealoma*.mp,kw. | 1902 | Advanced |  |  |  |
| 163 | pineoblastoma*.mp,kw. | 373 | Advanced |  |  |  |
| 164 | pineocytoma*.mp,kw. | 248 | Advanced |  |  |  |
| 165 | plasmacytom*.mp,kw. | 10302 | Advanced |  |  |  |
| 166 | (polycythem* adj2 vera?).mp,kw. | 7368 | Advanced |  |  |  |
| 167 | prolactinom*.mp,kw. | 4007 | Advanced |  |  |  |
| 168 | retinoblastom*.mp,kw. | 21098 | Advanced |  |  |  |
| 169 | rhabdoid*.mp,kw. | 2400 | Advanced |  |  |  |
| 170 | rhabdomyom*.mp,kw. | 1668 | Advanced |  |  |  |
| 171 | rhabdomyosarcom*.mp,kw. | 13657 | Advanced |  |  |  |
| 172 | sarcom*.mp,kw. | 121662 | Advanced |  |  |  |
| 173 | seminoma*.mp,kw. | 7271 | Advanced |  |  |  |
| 174 | Sertoli- Leydig.mp,kw. | 1138 | Advanced |  |  |  |
| 175 | somatostatinoma*.mp,kw. | 446 | Advanced |  |  |  |
| 176 | somatotrophinom*.mp,kw. | 75 | Advanced |  |  |  |
| 177 | struma ovarii*.mp,kw. | 620 | Advanced |  |  |  |
| 178 | thecoma*.mp,kw. | 1046 | Advanced |  |  |  |
| 179 | teratocarcin*.mp,kw. | 2798 | Advanced |  |  |  |
| 180 | teratoma*.mp,kw. | 20974 | Advanced |  |  |  |
| 181 | thymom*.mp,kw. | 10420 | Advanced |  |  |  |
| 182 | trophoblast*.mp,kw. | 24181 | Advanced |  |  |  |
| 183 | vipoma*.mp,kw. | 603 | Advanced |  |  |  |
| 184 | wilms*.mp. | 12311 | Advanced |  |  |  |
| 185 | or/1-184 | 4375119 | Advanced |  |  |  |
| 186 | Immunotherapy/ | 44006 | Advanced |  |  |  |
| 187 | Antibodies, Monoclonal/ | 187884 | Advanced |  |  |  |
| 188 | Antineoplastic Agents, Immunological/ | 4235 | Advanced |  |  |  |
| 189 | Ipilimumab/ | 1739 | Advanced |  |  |  |
| 190 | Nivolumab/ | 2095 | Advanced |  |  |  |
| 191 | (checkpoint? adj3 inhibitor?).mp,kw. | 5362 | Advanced |  |  |  |
| 192 | (check-point? adj3 inhibitor?).mp,kw. | 181 | Advanced |  |  |  |
| 193 | atezolizumab*.mp,kw. | 571 | Advanced |  |  |  |
| 194 | mpdl 3280a.mp,kw. | 3 | Advanced |  |  |  |
| 195 | mpdl3280a.mp,kw. | 34 | Advanced |  |  |  |
| 196 | rg 7446.mp,kw. | 0 | Advanced |  |  |  |
| 197 | rg7446.mp,kw. | 1 | Advanced |  |  |  |
| 198 | tec?ntriq*.mp,kw. | 16 | Advanced |  |  |  |
| 199 | 1380723-44-3.rn. | 0 | Advanced |  |  |  |
| 200 | ipilimumab*.mp,kw. | 2485 | Advanced |  |  |  |
| 201 | bms 734016.mp,kw. | 3 | Advanced |  |  |  |
| 202 | bms734016.mp,kw. | 1 | Advanced |  |  |  |
| 203 | "mdx 010".mp,kw. | 23 | Advanced |  |  |  |
| 204 | mdx010.mp,kw. | 8 | Advanced |  |  |  |
| 205 | mdx 101.mp,kw. | 3 | Advanced |  |  |  |
| 206 | mdx101.mp,kw. | 0 | Advanced |  |  |  |
| 207 | mdx ctla 4.mp,kw. | 2 | Advanced |  |  |  |
| 208 | mdx ctla4.mp,kw. | 2 | Advanced |  |  |  |
| 209 | strentarga*.mp,kw. | 0 | Advanced |  |  |  |
| 210 | yervoy*.mp,kw. | 43 | Advanced |  |  |  |
| 211 | 477202-00-9.rn. | 0 | Advanced |  |  |  |
| 212 | nivolumab*.mp,kw. | 2903 | Advanced |  |  |  |
| 213 | bms 936558.mp,kw. | 15 | Advanced |  |  |  |
| 214 | bms936558.mp,kw. | 0 | Advanced |  |  |  |
| 215 | mdx 1106.mp,kw. | 3 | Advanced |  |  |  |
| 216 | mdx1106.mp,kw. | 0 | Advanced |  |  |  |
| 217 | ono 4538.mp,kw. | 9 | Advanced |  |  |  |
| 218 | ono4538.mp,kw. | 0 | Advanced |  |  |  |
| 219 | opdivo*.mp,kw. | 46 | Advanced |  |  |  |
| 220 | 946414-94-4.rn. | 0 | Advanced |  |  |  |
| 221 | pembrolizumab*.mp,kw. | 2131 | Advanced |  |  |  |
| 222 | keytruda*.mp,kw. | 45 | Advanced |  |  |  |
| 223 | lambrolizumab*.mp,kw. | 16 | Advanced |  |  |  |
| 224 | mk 3475.mp,kw. | 34 | Advanced |  |  |  |
| 225 | mk3475.mp,kw. | 2 | Advanced |  |  |  |
| 226 | 1374853-91-4.rn. | 0 | Advanced |  |  |  |
| 227 | ticilimumab*.mp,kw. | 6 | Advanced |  |  |  |
| 228 | cp 675 206.mp,kw. | 21 | Advanced |  |  |  |
| 229 | cp675 206.mp,kw. | 0 | Advanced |  |  |  |
| 230 | cp675206.mp,kw. | 0 | Advanced |  |  |  |
| 231 | tremelimumab*.mp,kw. | 211 | Advanced |  |  |  |
| 232 | 745013-59-6.rn. | 0 | Advanced |  |  |  |
| 233 | or/186-232 | 232233 | Advanced |  |  |  |
| 234 | Patient Reported Outcome Measures/ | 5118 | Advanced |  |  |  |
| 235 | Patient Outcome Assessment/ | 4510 | Advanced |  |  |  |
| 236 | Data Collection/ | 88888 | Advanced |  |  |  |
| 237 | "Surveys and Questionnaires"/ | 452282 | Advanced |  |  |  |
| 238 | Patient Health Questionnaire/ | 300 | Advanced |  |  |  |
| 239 | Self Report/ | 30678 | Advanced |  |  |  |
| 240 | Health Status/ | 79893 | Advanced |  |  |  |
| 241 | exp Health Status Indicators/ | 299034 | Advanced |  |  |  |
| 242 | (patient? adj3 report* adj3 outcome?).mp,kw. | 17014 | Advanced |  |  |  |
| 243 | (patient? adj3 assess* adj3 outcome?).mp,kw. | 8472 | Advanced |  |  |  |
| 244 | (patient? adj3 report* adj3 treatment related*).mp,kw. | 102 | Advanced |  |  |  |
| 245 | (patient? adj3 report* adj3 adverse*).mp,kw. | 2251 | Advanced |  |  |  |
| 246 | (patient? adj3 report* adj3 side-effec*).mp,kw. | 1005 | Advanced |  |  |  |
| 247 | (patient? adj3 report* adj3 health status*).mp,kw. | 300 | Advanced |  |  |  |
| 248 | PROs.mp,kw. | 8696 | Advanced |  |  |  |
| 249 | (self adj1 report*).mp,kw. | 137833 | Advanced |  |  |  |
| 250 | selfreport*.mp,kw. | 124 | Advanced |  |  |  |
| 251 | (survival* adj3 report*).mp,kw. | 5702 | Advanced |  |  |  |
| 252 | ("quality of life" adj3 report*).mp,kw. | 5072 | Advanced |  |  |  |
| 253 | ("quality of life" adj3 relat*).mp,kw. | 42977 | Advanced |  |  |  |
| 254 | HRQOL?.mp,kw. | 13307 | Advanced |  |  |  |
| 255 | WHOQOL*.mp,kw. | 2261 | Advanced |  |  |  |
| 256 | QoL-Brief.mp,kw. | 17 | Advanced |  |  |  |
| 257 | (survival* adj3 data*).mp,kw. | 13516 | Advanced |  |  |  |
| 258 | ("quality of life" adj3 data*).mp,kw. | 2808 | Advanced |  |  |  |
| 259 | ("real-world" adj3 data*).mp,kw. | 3228 | Advanced |  |  |  |
| 260 | survey*.mp,kw. | 897533 | Advanced |  |  |  |
| 261 | questionnaire*.mp,kw. | 648160 | Advanced |  |  |  |
| 262 | or/234-261 | 1490040 | Advanced |  |  |  |
| 263 | 185 and 233 and 262 | 2513 | Advanced |  |  |  |
| 264 | limit 263 to yr="2003 -Current" | 2192 | Advanced |  |  |  |

**Appendix B**

**
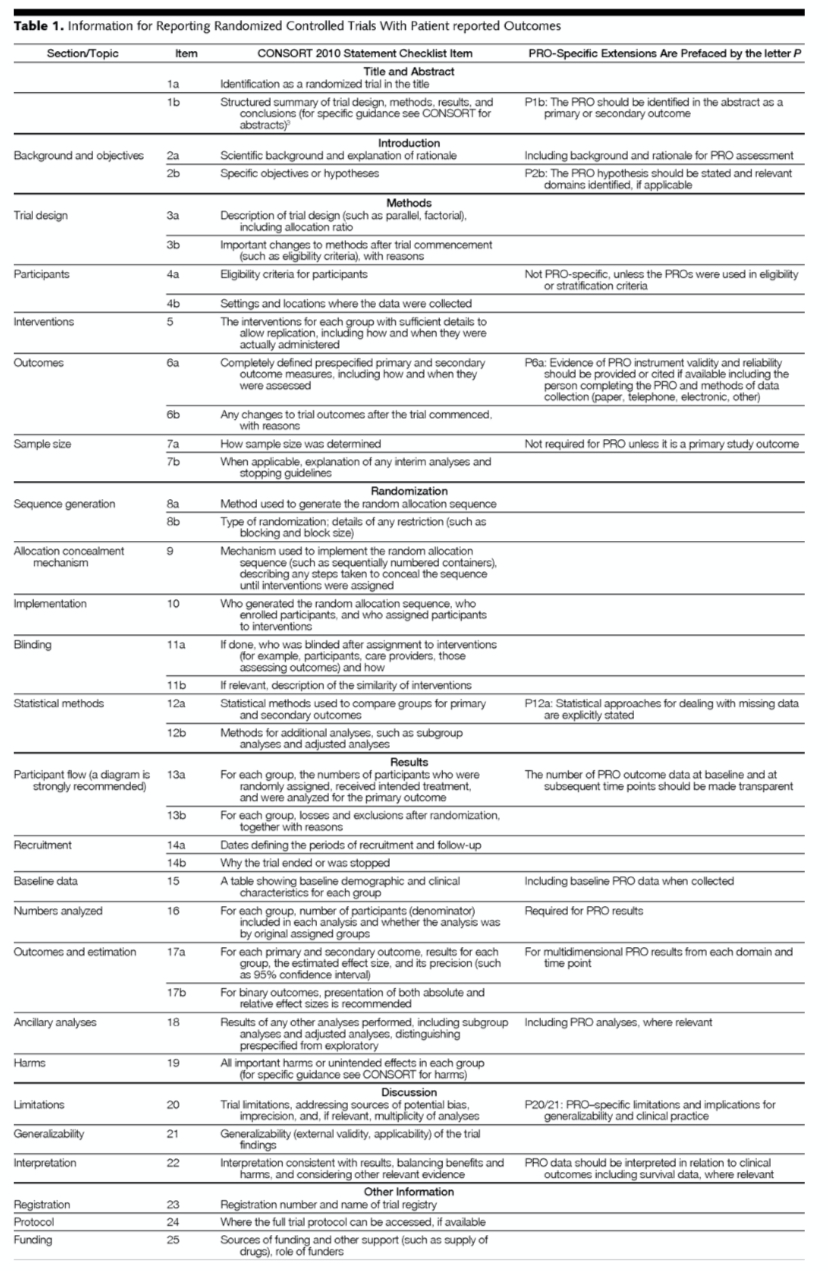
**

Reproduced from Reporting of Patient-Reported outcomes in Randomized Trials: The CONSORT PRO Extension. JAMA, February 27, 2013_Vol309(8) Copyright © 2013 American Medical association. All rights reserved.
