# Supplementary material for: Quality of patient‐reported outcomes in oncology clinical trials using immune checkpoint inhibitors: A systematic review
Source: Cancer Med. 2021 Jun 29;10(15):5031–40. doi: 10.1002/cam4.4086 (PMC8335827; doi:10.1002/cam4.4086)
Supplement: Supplementary file 2 — Supplementary Material [file CAM4-10-5031-s002.docx]

Appendix

References for the 33 clinical trials involved in the analysis

1. Antonia SJ, Villegas A, Daniel D, Vicente D, Murakami S, Hui R, et al. Durvalumab after Chemoradiotherapy in Stage III Non-Small-Cell Lung Cancer. N Engl J Med. 2017;377(20):1919-29.

2. Ascierto PA, Del Vecchio M, Robert C, Mackiewicz A, Chiarion-Sileni V, Arance A, et al. Ipilimumab 10 mg/kg versus ipilimumab 3 mg/kg in patients with unresectable or metastatic melanoma: a randomised, double-blind, multicentre, phase 3 trial. Lancet Oncol. 2017;18(5):611-22.

3. Atkins MB, Rini BI, Motzer RJ, Powles T, McDermott DF, Suarez C, et al. Patient-Reported Outcomes from the Phase III Randomized IMmotion151 Trial: Atezolizumab + Bevacizumab versus Sunitinib in Treatment-Naive Metastatic Renal Cell Carcinoma. Clin Cancer Res. 2020;26(11):2506-14.

4. Barlesi F, Garon EB, Kim DW, Felip E, Han JY, Kim JH, et al. Health-Related Quality of Life in KEYNOTE-010: a Phase II/III Study of Pembrolizumab Versus Docetaxel in Patients With Previously Treated Advanced, Programmed Death Ligand 1-Expressing NSCLC. J Thorac Oncol. 2019;14(5):793-801.

5. Bauml JM, Mick R, Ciunci C, Aggarwal C, Davis C, Evans T, et al. Pembrolizumab After Completion of Locally Ablative Therapy for Oligometastatic Non-Small Cell Lung Cancer: A Phase 2 Trial. JAMA Oncol. 2019.

6. Bellmunt J, de Wit R, Vaughn DJ, Fradet Y, Lee JL, Fong L, et al. Pembrolizumab as Second-Line Therapy for Advanced Urothelial Carcinoma. N Engl J Med. 2017;376(11):1015-26.

7. Bordoni R, Ciardiello F, von Pawel J, Cortinovis D, Karagiannis T, Ballinger M, et al. Patient-Reported Outcomes in OAK: A Phase III Study of Atezolizumab Versus Docetaxel in Advanced Non-Small-cell Lung Cancer. Clin Lung Cancer. 2018;19(5):441-9 e4.

8. Borghaei H, Paz-Ares L, Horn L, Spigel DR, Steins M, Ready NE, et al. Nivolumab versus Docetaxel in Advanced Nonsquamous Non-Small-Cell Lung Cancer. N Engl J Med. 2015;373(17):1627-39.

9. Brahmer J, Reckamp KL, Baas P, Crino L, Eberhardt WE, Poddubskaya E, et al. Nivolumab versus Docetaxel in Advanced Squamous-Cell Non-Small-Cell Lung Cancer. N Engl J Med. 2015;373(2):123-35.

10. Brahmer JR, Rodriguez-Abreu D, Robinson AG, Hui R, Csoszi T, Fulop A, et al. Health-related quality-of-life results for pembrolizumab versus chemotherapy in advanced, PD-L1-positive NSCLC (KEYNOTE-024): a multicentre, international, randomised, open-label phase 3 trial. Lancet Oncol. 2017;18(12):1600-9.

11. Cella D, Grunwald V, Escudier B, Hammers HJ, George S, Nathan P, et al. Patient-reported outcomes of patients with advanced renal cell carcinoma treated with nivolumab plus ipilimumab versus sunitinib (CheckMate 214): a randomised, phase 3 trial. Lancet Oncol. 2019;20(2):297-310.

12. Cella D, Grunwald V, Nathan P, Doan J, Dastani H, Taylor F, et al. Quality of life in patients with advanced renal cell carcinoma given nivolumab versus everolimus in CheckMate 025: a randomised, open-label, phase 3 trial. Lancet Oncol. 2016;17(7):994-1003.

13. Chen R, Zinzani PL, Fanale MA, Armand P, Johnson NA, Brice P, et al. Phase II Study of the Efficacy and Safety of Pembrolizumab for Relapsed/Refractory Classic Hodgkin Lymphoma. J Clin Oncol. 2017;35(19):2125-32.

14. Coens C, Suciu S, Chiarion-Sileni V, Grob JJ, Dummer R, Wolchok JD, et al. Health-related quality of life with adjuvant ipilimumab versus placebo after complete resection of high-risk stage III melanoma (EORTC 18071): secondary outcomes of a multinational, randomised, double-blind, phase 3 trial. Lancet Oncol. 2017;18(3):393-403.

15. Eggermont AMM, Chiarion-Sileni V, Grob JJ, Dummer R, Wolchok JD, Schmidt H, et al. Adjuvant ipilimumab versus placebo after complete resection of stage III melanoma: long-term follow-up results of the European Organisation for Research and Treatment of Cancer 18071 double-blind phase 3 randomised trial. Eur J Cancer. 2019;119:1-10.

16. Ferris RL, Blumenschein G, Jr., Fayette J, Guigay J, Colevas AD, Licitra L, et al. Nivolumab for Recurrent Squamous-Cell Carcinoma of the Head and Neck. N Engl J Med. 2016;375(19):1856-67.

17. Gandhi L, Rodriguez-Abreu D, Gadgeel S, Esteban E, Felip E, De Angelis F, et al. Pembrolizumab plus Chemotherapy in Metastatic Non-Small-Cell Lung Cancer. N Engl J Med. 2018;378(22):2078-92.

18. Garassino MC, Gadgeel S, Esteban E, Felip E, Speranza G, Domine M, et al. Patient-reported outcomes following pembrolizumab or placebo plus pemetrexed and platinum in patients with previously untreated, metastatic, non-squamous non-small-cell lung cancer (KEYNOTE-189): a multicentre, double-blind, randomised, placebo-controlled, phase 3 trial. Lancet Oncol. 2020;21(3):387-97.

19. Harrington KJ, Ferris RL, Blumenschein G, Jr., Colevas AD, Fayette J, Licitra L, et al. Nivolumab versus standard, single-agent therapy of investigator's choice in recurrent or metastatic squamous cell carcinoma of the head and neck (CheckMate 141): health-related quality-of-life results from a randomised, phase 3 trial. Lancet Oncol. 2017;18(8):1104-15.

20. Hellmann MD, Paz-Ares L, Bernabe Caro R, Zurawski B, Kim SW, Carcereny Costa E, et al. Nivolumab plus Ipilimumab in Advanced Non-Small-Cell Lung Cancer. N Engl J Med. 2019;381(21):2020-31.

21. Herbst RS, Baas P, Kim DW, Felip E, Perez-Gracia JL, Han JY, et al. Pembrolizumab versus docetaxel for previously treated, PD-L1-positive, advanced non-small-cell lung cancer (KEYNOTE-010): a randomised controlled trial. Lancet. 2016;387(10027):1540-50.

22. Hodi FS, O'Day SJ, McDermott DF, Weber RW, Sosman JA, Haanen JB, et al. Improved survival with ipilimumab in patients with metastatic melanoma. N Engl J Med. 2010;363(8):711-23.

23. Horn L, Mansfield AS, Szczesna A, Havel L, Krzakowski M, Hochmair MJ, et al. First-Line Atezolizumab plus Chemotherapy in Extensive-Stage Small-Cell Lung Cancer. N Engl J Med. 2018;379(23):2220-9.

24. Hui R, Ozguroglu M, Villegas A, Daniel D, Vicente D, Murakami S, et al. Patient-reported outcomes with durvalumab after chemoradiotherapy in stage III, unresectable non-small-cell lung cancer (PACIFIC): a randomised, controlled, phase 3 study. Lancet Oncol. 2019;20(12):1670-80.

25. Kato K, Cho BC, Takahashi M, Okada M, Lin CY, Chin K, et al. Nivolumab versus chemotherapy in patients with advanced oesophageal squamous cell carcinoma refractory or intolerant to previous chemotherapy (ATTRACTION-3): a multicentre, randomised, open-label, phase 3 trial. Lancet Oncol. 2019;20(11):1506-17.

26. Kaufman HL, Hunger M, Hennessy M, Schlichting M, Bharmal M. Nonprogression with avelumab treatment associated with gains in quality of life in metastatic Merkel cell carcinoma. Future Oncol. 2018;14(3):255-66.

27. Larkin J, Minor D, D'Angelo S, Neyns B, Smylie M, Miller WH, Jr., et al. Overall Survival in Patients With Advanced Melanoma Who Received Nivolumab Versus Investigator's Choice Chemotherapy in CheckMate 037: A Randomized, Controlled, Open-Label Phase III Trial. J Clin Oncol. 2018;36(4):383-90.

28. Long GV, Atkinson V, Ascierto PA, Robert C, Hassel JC, Rutkowski P, et al. Effect of nivolumab on health-related quality of life in patients with treatment-naive advanced melanoma: results from the phase III CheckMate 066 study. Ann Oncol. 2016;27(10):1940-6.

29. Mansfield AS, Kazarnowicz A, Karaseva N, Sanchez A, De Boer R, Andric Z, et al. Safety and patient-reported outcomes of atezolizumab, carboplatin, and etoposide in extensive-stage small-cell lung cancer (IMpower133): a randomized phase I/III trial. Ann Oncol. 2020;31(2):310-7.

30. Mazieres J, Kowalski D, Luft A, Vicente D, Tafreshi A, Gumus M, et al. Health-Related Quality of Life With Carboplatin-Paclitaxel or nab-Paclitaxel With or Without Pembrolizumab in Patients With Metastatic Squamous Non-Small-Cell Lung Cancer. J Clin Oncol. 2020;38(3):271-80.

31. McGregor BA, McKay RR, Braun DA, Werner L, Gray K, Flaifel A, et al. Results of a Multicenter Phase II Study of Atezolizumab and Bevacizumab for Patients With Metastatic Renal Cell Carcinoma With Variant Histology and/or Sarcomatoid Features. J Clin Oncol. 2020;38(1):63-70.

32. Mirau PA, Kearns DR. Sequence and conformational effects on imino proton exchange in A.T- and A.U-containing DNA and RNA duplexes. Biopolymers. 1985;24(4):711-24.

33. Motzer RJ, Escudier B, McDermott DF, George S, Hammers HJ, Srinivas S, et al. Nivolumab versus Everolimus in Advanced Renal-Cell Carcinoma. N Engl J Med. 2015;373(19):1803-13.

34. Motzer RJ, Tannir NM, McDermott DF, Aren Frontera O, Melichar B, Choueiri TK, et al. Nivolumab plus Ipilimumab versus Sunitinib in Advanced Renal-Cell Carcinoma. N Engl J Med. 2018;378(14):1277-90.

35. Naumann RW, Hollebecque A, Meyer T, Devlin MJ, Oaknin A, Kerger J, et al. Safety and Efficacy of Nivolumab Monotherapy in Recurrent or Metastatic Cervical, Vaginal, or Vulvar Carcinoma: Results From the Phase I/II CheckMate 358 Trial. J Clin Oncol. 2019;37(31):2825-34.

36. Overman MJ, Lonardi S, Wong KYM, Lenz HJ, Gelsomino F, Aglietta M, et al. Durable Clinical Benefit With Nivolumab Plus Ipilimumab in DNA Mismatch Repair-Deficient/Microsatellite Instability-High Metastatic Colorectal Cancer. J Clin Oncol. 2018;36(8):773-9.

37. Overman MJ, McDermott R, Leach JL, Lonardi S, Lenz HJ, Morse MA, et al. Nivolumab in patients with metastatic DNA mismatch repair-deficient or microsatellite instability-high colorectal cancer (CheckMate 142): an open-label, multicentre, phase 2 study. Lancet Oncol. 2017;18(9):1182-91.

38. Paz-Ares L, Luft A, Vicente D, Tafreshi A, Gumus M, Mazieres J, et al. Pembrolizumab plus Chemotherapy for Squamous Non-Small-Cell Lung Cancer. N Engl J Med. 2018;379(21):2040-51.

39. Petrella TM, Robert C, Richtig E, Miller WH, Jr., Masucci GV, Walpole E, et al. Patient-reported outcomes in KEYNOTE-006, a randomised study of pembrolizumab versus ipilimumab in patients with advanced melanoma. Eur J Cancer. 2017;86:115-24.

40. Powles T, Duran I, van der Heijden MS, Loriot Y, Vogelzang NJ, De Giorgi U, et al. Atezolizumab versus chemotherapy in patients with platinum-treated locally advanced or metastatic urothelial carcinoma (IMvigor211): a multicentre, open-label, phase 3 randomised controlled trial. Lancet. 2018;391(10122):748-57.

41. Reck M, Brahmer J, Bennett B, Taylor F, Penrod JR, DeRosa M, et al. Evaluation of health-related quality of life and symptoms in patients with advanced non-squamous non-small cell lung cancer treated with nivolumab or docetaxel in CheckMate 057. Eur J Cancer. 2018;102:23-30.

42. Reck M, Rodriguez-Abreu D, Robinson AG, Hui R, Csoszi T, Fulop A, et al. Pembrolizumab versus Chemotherapy for PD-L1-Positive Non-Small-Cell Lung Cancer. N Engl J Med. 2016;375(19):1823-33.

43. Reck M, Schenker M, Lee KH, Provencio M, Nishio M, Lesniewski-Kmak K, et al. Nivolumab plus ipilimumab versus chemotherapy as first-line treatment in advanced non-small-cell lung cancer with high tumour mutational burden: patient-reported outcomes results from the randomised, open-label, phase III CheckMate 227 trial. Eur J Cancer. 2019;116:137-47.

44. Reck M, Taylor F, Penrod JR, DeRosa M, Morrissey L, Dastani H, et al. Impact of Nivolumab versus Docetaxel on Health-Related Quality of Life and Symptoms in Patients with Advanced Squamous Non-Small Cell Lung Cancer: Results from the CheckMate 017 Study. J Thorac Oncol. 2018;13(2):194-204.

45. Revicki DA, van den Eertwegh AJ, Lorigan P, Lebbe C, Linette G, Ottensmeier CH, et al. Health related quality of life outcomes for unresectable stage III or IV melanoma patients receiving ipilimumab treatment. Health Qual Life Outcomes. 2012;10:66.

46. Ribas A, Puzanov I, Dummer R, Schadendorf D, Hamid O, Robert C, et al. Pembrolizumab versus investigator-choice chemotherapy for ipilimumab-refractory melanoma (KEYNOTE-002): a randomised, controlled, phase 2 trial. Lancet Oncol. 2015;16(8):908-18.

47. Rini BI, Powles T, Atkins MB, Escudier B, McDermott DF, Suarez C, et al. Atezolizumab plus bevacizumab versus sunitinib in patients with previously untreated metastatic renal cell carcinoma (IMmotion151): a multicentre, open-label, phase 3, randomised controlled trial. Lancet. 2019;393(10189):2404-15.

48. Rittmeyer A, Barlesi F, Waterkamp D, Park K, Ciardiello F, von Pawel J, et al. Atezolizumab versus docetaxel in patients with previously treated non-small-cell lung cancer (OAK): a phase 3, open-label, multicentre randomised controlled trial. Lancet. 2017;389(10066):255-65.

49. Robert C, Long GV, Brady B, Dutriaux C, Maio M, Mortier L, et al. Nivolumab in previously untreated melanoma without BRAF mutation. N Engl J Med. 2015;372(4):320-30.

50. Robert C, Schachter J, Long GV, Arance A, Grob JJ, Mortier L, et al. Pembrolizumab versus Ipilimumab in Advanced Melanoma. N Engl J Med. 2015;372(26):2521-32.

51. Schadendorf D, Dummer R, Hauschild A, Robert C, Hamid O, Daud A, et al. Health-related quality of life in the randomised KEYNOTE-002 study of pembrolizumab versus chemotherapy in patients with ipilimumab-refractory melanoma. Eur J Cancer. 2016;67:46-54.

52. Schadendorf D, Larkin J, Wolchok J, Hodi FS, Chiarion-Sileni V, Gonzalez R, et al. Health-related quality of life results from the phase III CheckMate 067 study. Eur J Cancer. 2017;82:80-91.

53. Vaughn DJ, Bellmunt J, Fradet Y, Lee JL, Fong L, Vogelzang NJ, et al. Health-Related Quality-of-Life Analysis From KEYNOTE-045: A Phase III Study of Pembrolizumab Versus Chemotherapy for Previously Treated Advanced Urothelial Cancer. J Clin Oncol. 2018;36(16):1579-87.

54. Weber J, Mandala M, Del Vecchio M, Gogas HJ, Arance AM, Cowey CL, et al. Adjuvant Nivolumab versus Ipilimumab in Resected Stage III or IV Melanoma. N Engl J Med. 2017;377(19):1824-35.

55. Wolchok JD, Chiarion-Sileni V, Gonzalez R, Rutkowski P, Grob JJ, Cowey CL, et al. Overall Survival with Combined Nivolumab and Ipilimumab in Advanced Melanoma. N Engl J Med. 2017;377(14):1345-56.

56. Zinzani PL, Santoro A, Gritti G, Brice P, Barr PM, Kuruvilla J, et al. Nivolumab Combined With Brentuximab Vedotin for Relapsed/Refractory Primary Mediastinal Large B-Cell Lymphoma: Efficacy and Safety From the Phase II CheckMate 436 Study. J Clin Oncol. 2019;37(33):3081-9.
